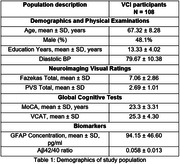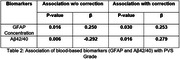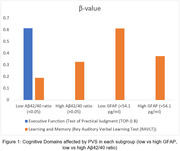# Association of Blood‐Based Biomarkers: GFAP, Amyloid 42/40 and Perivascular Spaces in Relation to Cognitive Domains in Vascular Cognitive Impairment Participants

**DOI:** 10.1002/alz.092210

**Published:** 2025-01-09

**Authors:** Jia Dong James Wang, Yi Jin Leow, Ashwati Vipin, Gurveen Kaur Sandhu, Pricilia Tanoto, Mohammed Adnan Azam, Fatin Zahra Zailan, Faith Phemie Hui En Lee, Smriti Ghildiyal, Shan Yao Liew, Isabelle Yu Zhen Tan, Nagaendran Kandiah

**Affiliations:** ^1^ Lee Kong Chian School of Medicine, Nanyang Technological University, Singapore Singapore

## Abstract

**Background:**

Perivascular spaces (PVS) are fluid‐filled spaces in the brain, hypothesized in promoting clearance of metabolites implicated in dementia through glymphatic system drainage. PVS are classified according to Grades (0‐4). Blood‐based biomarkers including Glial fibrillary acidic protein (GFAP), Amyloid β 42/40 (Aβ42/40) ratio have shown promise in diagnosing and prognosing dementia.

However, the association between blood‐based biomarkers and PVS remains relatively unexplored in Vascular Cognitive Impairment (VCI) participants. Thus, this study aims to characterize association between blood‐based biomarkers and PVS in relation to cognitive domains in VCI participants.

**Method:**

Participants from the Biomarkers and Cognition Study, Singapore were included and VCI was defined in participants with confluent white matter hyperintensities (WMH), >1 lacunae and Montreal Cognitive Assessment (MoCA) score <26.

108 participants (mean age 67.3, education years 13.3, 51.9% females) were included.

Normality tests, multivariate ordinal correlation analysis was performed to understand association between blood‐based biomarkers and PVS grade, with correction for age, diastolic blood pressure and WMH.

Aβ42/40 and GFAP ratio values of 0.05 and 54.1pg/ml were utilized to segregate VCI participants to different subgroups (high vs low levels) and their association with cognitive domain performance was assessed.

**Result:**

Higher GFAP (p=0.026) and lower Aβ42/40 ratio (p=0.049) was associated with higher PVS grade.

In the low Aβ42/40 ratio subgroup, higher PVS grade was most significantly associated with executive function impairment (p = 0.045, β = 0.612). For the subgroups of high Aβ42/40 ratio (p=0.017, β = 0.325), low GFAP (p = 0.027, β = 0.610), high GFAP (p = 0.006, β = 0.375), higher PVS grade was most significantly associated with learning and memory impairment.

**Conclusion:**

These results demonstrate that low Aβ42/40 ratio, high GFAP levels were associated with higher PVS grade in VCI participants, suggesting their utility as markers for VCI.

Furthermore, higher PVS Grade in VCI participants with low Aβ42/40 ratio were associated with executive function impairment compared to other subgroups which were associated with learning and memory impairment. This could be an important clinical marker when examining patients with high PVS Grade as it could be associated with a more sinister disease progression.